# Effects of Supplementation With Genistein on the Progression of Cognitive Decline in Patients With Mild Cognitive Impairment Due to Alzheimer Disease: Protocol for a Randomized Controlled Trial

**DOI:** 10.2196/98087

**Published:** 2026-11-09

**Authors:** Francisco Javier López Román, Manuel Sebastián, Silvia Pérez-Piñero, Ingrid Hernández-Manzano, Olga Laosa-Zafra, Leocadio Rodríguez-Mañas, Francisco José Tarazona-Santabalbina, Consuelo Borrás, José Viña

**Affiliations:** 1 Department of Exercise Physiology, Faculty of Medicine, Universidad Católica de Murcia, Avenida de los Jerónimos, Murcia, 30107, Spain, +34 968278157 ext 157; 2 Primary Care Research Group, Instituto Murciano de Investigación Biosanitaria, Murcia, Spain; 3 Faculty of Medicine, Universidad Católica de Murcia, Av. de los Jerónimos, 135, Guadalupe (Murcia), 30107, Spain, +34 968 278 157; 4 Foundation for Biomedical Research, Hospital Universitario de Getafe, Madrid, Spain; 5 Department of Geriatrics, Hospital Universitario de Getafe, Madrid, Spain; 6 Spanish Society of Geriatrics and Gerontology (SEGG), Madrid, Spain; 7 Hospital de La Ribera, Alzira, Spain; 8 Department of Physiology, Faculty of Medicine, Universitat de València, València, Spain; 9 Department of Geroscience, Universidad Católica de Murcia, Murcia, Spain

**Keywords:** Alzheimer disease, amyloid-beta, dementia, genistein, mild cognitive impairment, neuropsychological tests, randomized controlled trial, soy isoflavones

## Abstract

**Background:**

Mild cognitive impairment (MCI) may represent a prodromal stage of Alzheimer disease, but interventions to delay progression remain limited. Genistein is a phytoestrogenic isoflavone with potential effects on several pathways implicated in Alzheimer disease. We previously showed that genistein reverses cognitive decline and lowers brain amyloid β (Aβ) in the Aβ precursor protein/presenilin 1 double transgenic (AβPP/PS1) model of Alzheimer disease.

**Objective:**

The study aimed to evaluate whether oral genistein supplementation affects cognitive decline in patients with MCI due to Alzheimer disease, compared with a placebo, and to assess its effects on brain amyloid burden and plasma biomarkers.

**Methods:**

This multicenter, randomized, double-blind, placebo-controlled trial will include adults aged 50 years or older with MCI due to Alzheimer disease. Participants will be randomized 1:1 to genistein (200 mg/day) or a maltodextrin-based placebo for 18 months. The primary outcome is change in the Integrated Alzheimer’s Disease Rating Scale (iADRS) score. Secondary outcomes include other neuropsychological measures, amyloid burden measured by ^18^F-flutemetamol positron emission tomography, plasma Alzheimer disease biomarkers, and safety. Outcomes will be assessed at baseline, midstudy, and study completion. Analyses are planned in the intention-to-treat and per-protocol populations using repeated-measures analysis of variance, with time as the within-participant factor, treatment group as the between-participant factor, and study site as a covariate.

**Results:**

Funding for the study began in January 2024 and was provided by Trugon Investigaciones (AIE) through a grant to the Chair of Geroscience at Universidad Católica de Murcia. Data collection began in July 2026 and is projected to conclude in January 2029, consistent with the estimated 30-month study duration. Enrollment is expected to last approximately 9 months. As of August 5, 2026, a total of 4 participants had been recruited. Data analysis has not begun and will start after the final participant completes follow-up. Results are expected to be published in late 2029.

**Conclusions:**

This trial will provide evidence on whether genistein supplementation may help slow cognitive decline and reduce amyloid burden in patients with MCI due to Alzheimer disease.

## Introduction

### Background

Mild cognitive impairment (MCI) represents an intermediate stage between normal cognitive aging and established dementia. It is characterized by an objective decline in one or more cognitive domains, particularly memory, executive function, or language, that does not significantly interfere with the individual’s functional independence [1]. Longitudinal studies have shown that approximately 10% to 15% of patients with MCI progress to dementia each year, with Alzheimer disease being the most frequent cause [2]. However, this progression is not inevitable, as nearly 30% of cases remain stable, and about 15% show improvement or revert to normal or near-normal cognition [3]. This heterogeneity in the progression of MCI to clinically overt dementia has prompted the search for predictive biomarkers and, in particular, early interventions that may modify the natural course of the disease.

From a pathophysiological perspective, MCI associated with Alzheimer disease shares the same neurobiological substrates as established dementia, although to a lesser degree, including the accumulation of amyloid-β (Aβ) plaques, neurofibrillary tangles of hyperphosphorylated τ, oxidative stress, synaptic loss, and hippocampal atrophy [4]. The therapeutic window of MCI is particularly attractive because neuroprotective strategies may be more effective during this prodromal phase or preclinical stage, when neuronal damage is still limited [5]. However, over the past 20 years, advances in the search for effective treatments to modify the progression of Alzheimer disease have been modest. The clinical efficacy of treatments based on the intravenous administration of monoclonal antibodies targeting specific proteins involved in the pathophysiology of the disease, such as Aβ and phosphorylated τ, has been generally disappointing. Although antiamyloid therapies may slow disease progression, serious side effects such as amyloid‐related imaging abnormalities, edema, and hemorrhage are a matter of concern [6]. Moreover, the requirement for intravenous drug administration over prolonged periods complicates adherence to treatment. Clinical trials of antiamyloid agents in patients with MCI have shown contradictory results, highlighting the need to explore the effectiveness of additional drugs with broader pharmacological actions and more favorable safety profiles [7].

In this context, genistein, a phytoestrogenic isoflavone abundant in soy products, has emerged as a promising candidate due to its multimodal pharmacological properties. In contrast to conventional drugs that act on a single target, genistein exerts simultaneous effects on multiple underlying pathogenic pathways of MCI and Alzheimer disease [8]. This compound modulates Aβ metabolism and clearance from the brain. It binds to the peroxisome proliferator-activated receptor-γ (PPARγ) subunit of the retinoid X receptor–PPARγ heterodimer dimer, thus activating apolipoprotein E (ApoE) production, which in turn stimulates Aβ clearance from the brain [1]. Moreover, it inhibits β-secretase (BACE1) activity and stimulates Aβ degradation by activating neprilysin [9,10]. It also has anti-τ properties, reducing abnormal τ phosphorylation via inhibition of kinases like GSK-3β (glycogen synthase kinase-3β) [11], as well as mitochondrial neuroprotection by increasing the expression of superoxide dismutase 2 (SOD2) and catalase, antioxidant enzymes that mitigate oxidative stress [12]. Moreover, genistein exhibits anti-inflammatory effects by suppressing microglial activation and the production of proinflammatory cytokines (interleukin-6 [IL-6] and tumor necrosis factor-α [TNFα]), which are dependent on nuclear factor-kB (NF-kB) [13]. The cognitive-potentiating effects of genistein may be associated with improvements in synaptic plasticity, mediated by the positive regulation of brain-derived neurotrophic factor (BDNF) and *N*-methyl-d-aspartate (NMDA) receptors [14].

### Rationale

Experimental studies have shown that genistein is useful to reverse the loss of cognition and pathological hallmarks in animal models of Alzheimer disease by targeting specific pathophysiological and signaling mechanisms involved in the neuropathology of the disease. In an Aβ precursor protein/presenilin 1 double transgenic (AβPP/PS1) mouse model, treatment with genistein resulted in a remarkable and rapid improvement in various parameters of cognition (such as hippocampal learning, recognition memory, implicit memory, and odor discrimination), which was associated with a lowering of Aβ levels in the brain, number and areas of amyloid plaques, and microglial reactivity [12]. This beneficial effect of genistein was mediated by a PPARγ-dependent increase in ApoE released from astrocytes [12]. In another rat model of Alzheimer disease, genistein showed neuroprotective effects by inhibiting the endoplasmic reticulum stress–mediated apoptotic pathway, reducing damage to hippocampal neurons and improving learning and memory [15]. Moreover, in streptozotocin (STZ)–treated male rats, a model of sporadic Alzheimer disease, high doses of genistein (150 mg/kg/day) normalized behavioral and biochemical deficits, with activation of autophagy appearing to be the primary mechanism [16]. Moreover, in vitro*,* neuronal models have shown the pleiotropic effects of genistein at intracellular and extracellular levels [17-19]. Taken together, this robust preclinical evidence of genistein’s pleiotropic effects on PPARγ supports the potential role of this naturally occurring compound in the therapeutic armamentarium for Alzheimer disease.

However, translation of experimental studies to humans is very limited and remains restricted to the preliminary results of a single clinical trial that we performed, in which 24 patients with prodromal Alzheimer disease were treated with 120 mg/day of genistein or a placebo for 24 months [20]. Aβ deposition was analyzed using ^18^F-flutemetamol uptake, and cognitive changes were assessed using a battery of neurocognitive tests. The main finding of the study was the lack of increase in Aβ uptake in the anterior cingulate gyrus in genistein-treated patients, compared with significant increases in the placebo group. Moreover, the results of neurocognitive tests showed an overall tendency toward improvement in the genistein group, reaching statistical significance in 2 of them (the dichotomized TAVEC [Aprendizaje Verbal España-Complutense Test] and Centil Rey Delayed Copy tests) vs placebo. A combined Alzheimer disease score, calculated from all 12 tests, showed a statistically significant improvement in individuals taking genistein compared with a placebo [20]. These preliminary positive findings in a small study population provide the basis for designing the present larger clinical trial with broader outcomes.

### Objectives

The primary objective is to assess the effects of dietary supplementation with genistein on the progression of cognitive decline in patients diagnosed with MCI due to Alzheimer disease, compared with a placebo, after 18 months of treatment, in relation to the following functions: (1) global assessment of dementia: evaluation of overall functionality and cognitive decline in patients with MCI; (2) assessment of global cognitive functions; (3) assessment of episodic memory; (4) assessment of the patient’s ability to perform activities of daily living (basic or instrumental); and (5) assessment of depressive emotional status.

Secondary objectives are the following: (1) to assess changes in neurological clinical manifestations; (2) to assess changes in Aβ load on brain neuroimaging; (3) to assess the modifications produced by the investigational product on serum biomarkers of Alzheimer disease; and (4) to assess the safety of the investigational product.

## Methods

### Study Design and Patients

The proposed study is a multicenter, randomized, double-blind, placebo-controlled clinical trial with 2 parallel arms (experimental and placebo) to be conducted at the outpatient clinics of the neurology or geriatrics departments of secondary and tertiary care hospitals in the Spanish National Health System across the country. The trial design follows the SPIRIT (Standard Protocol Items: Recommendations for Interventional Trials) 2025 guidelines (Checklist 1 [21]). The trial report will adhere to the CONSORT (Consolidated Standards of Reporting Trials) guidelines [22].

Participants are patients aged 50 years or older diagnosed with MCI due to Alzheimer disease according to the criteria of the National Institute on Aging and Alzheimer Association [23] who meet the eligibility criteria and provide written informed consent. The inclusion and exclusion criteria are summarized in Textbox 1. The enrollment period is expected to last approximately 9 months from the first study visit. The follow-up period will extend for 18 months, with an estimated total study duration of 30 months.

Textbox 1. **Eligibility criteria.**

**Inclusion criteria**

- Patients aged 50 years or older
- Diagnosis of mild cognitive impairment due to Alzheimer disease according to criteria of the National Institute on Aging and Alzheimer’s Association [24]
  - A Mini-Mental State Examination score between 22 and 30 inclusive (exceptions can be made for patients with fewer than 5 years of formal education).
  - Evidence of concern for cognitive change compared to the person’s previous level (complaint/subjective memory impairment within the past year for more than 6 months and/or confirmed by the informant and/or the physician).
  - Clinical dementia rating of 0.5, with a score of 0.5 in the memory domain.
  - Essentially preserved activities of daily living.

- Evidence of increased cortical amyloid as shown by ^18^F-flutemetamol positron emission tomography imaging.

**Exclusion criteria**

- Diagnosis of a neurological disease other than Alzheimer disease.
- Moderate depression using the Geriatric Depression Scale-15 with a score >8 or presence of any other severe psychiatric disorder.
- Treatment with an acetylcholinesterase inhibitor and/or memantine in the past 3 months.
- Use of isoflavones-containing supplements.
- Presence of hormone-dependent neoplasms.
- Diagnosis of significant cerebrovascular disease.
- Presence of any severe systemic disease preventing completion of the study.
- Refusal to provide the informed consent.

### Ethical Considerations

The study adheres to the principles of the Declaration of Helsinki and Good Clinical Practice guidelines. The study protocol was approved by the Clinical Research Ethics Committee of Hospital Universitario de Getafe, Madrid (Spain; code 25/144, approval date: February 3, 2026) and Hospital Universitario de La Ribera, Alzira, Valencia (Spain; code MULT030226, approval date: March 31, 2026). Written informed consent will be obtained from all participants. To safeguard privacy, study data will be processed in accordance with the Spanish Data Protection Law 3/2018 [25], using a deidentified database to ensure full confidentiality. The trial is registered at ClinicalTrials.gov (NCT07385937).

### Randomization and Blinding

A stratified randomization by center will be performed, with patients assigned in a 1:1 ratio to the experimental or placebo arms by a simple randomization procedure using the Epidat (version 4.2; Xunta de Galicia) software program. The randomization list will be maintained by a member of the research team who is independent of the study. The number of randomization codes allocated to each center will be determined by the anticipated patient recruitment. Recruitment will be competitive; therefore, if a center uses all of its assigned codes, additional codes will be issued from the general randomization list generated at the initiation of the study. After the randomization list is distributed to the centers, the principal investigator (PI) will be responsible for securely maintaining it and filing it in the investigator’s file, which will be kept at the research center.

This is a double-blind study aimed at minimizing bias, with patients and investigators blinded simultaneously to the patient’s treatment allocation. The investigational products will present the same organoleptic characteristics and will be labeled with the trial protocol number and each participant’s randomization code. Each eligible participant will be assigned a unique 5-character identification number: 2 letters for the center and 3 sequential digits corresponding to the assigned treatment number. In the case of a serious adverse event (AE) or an emergency requiring unblinding, the attending researcher must inform the PI, who will disclose the assigned treatment. All unblinding must be documented in the participant’s medical record and the electronic case report form.

### Patient Withdrawal and Discontinuation

Participation in the study is voluntary, and no compensation will be offered. Patients may withdraw from the study at any time, without the need to provide a reason and without incurring any personal disadvantage. Patients who discontinue the study will not be replaced. The criteria for withdrawal include the following: protocol deviations, occurrence of AEs, the investigator’s decision, the patient’s refusal to continue the study, and loss to follow-up.

### Intervention

#### Investigational Products

The experimental product is an encapsulated pure genistein powder extract, prepared by the Pharmacy Departments of Catholic University of Murcia (UCAM). The product was commercially acquired from the company South Sea Science Limited. UCAM has been exclusively responsible for the encapsulation process of the product to ensure proper blinding of the study, and the authors have no commercial associations with the intervention drug or its source.

Each capsule contains 100 mg of genistein, with a daily study dose of 200 mg, which is consistent with the safety, tolerability, and pharmacokinetics of single ascending doses of genistein reported in healthy volunteers [24]. Placebo capsules contain maltodextrin and are equal in shape and size to those of the investigational product. Patients will be instructed to take 2 capsules of the assigned product orally twice a day. It will be recommended to take each capsule about 30 minutes before breakfast and dinner, respectively, with a glass of water if needed. Participants will be advised to maintain their routine dietary habits and physical activity and not to initiate any drug treatment during the study unless fully justified and approved by the investigators.

#### Acquisition, Labeling, Storing, and Dispensing

The investigational products will be labeled in accordance with the Good Manufacturing Practice (GMP) guidelines for medicinal products for human and veterinary use. The label will include the batch number, the clinical trial reference code, the patients’ randomization code, the statement “for clinical trial use only,” the storage conditions, the shelf life (expiry date), and the statement “keep out of the reach of children.”

The investigational products will be available exclusively for this study and will remain under the responsibility of the PI at each study site for proper custody, storing under appropriate temperature and humidity conditions (at room temperature, preferably in a dry, cool place, and protected from direct sunlight), and dispensing to the study participants according to the randomization code list. Upon completion of the treatment period, the PI at each site will collect the returned capsules from participants and any remaining investigational products. Capsules returned by participants will be counted to assess compliance with treatment, defined as consumption of at least 80% of the assigned product.

### Study Procedures

The study includes a screening (selection) visit (V0) and 7 study visits (V1-V7) scheduled along the 540 days of the study period, with a baseline visit (V1) and follow-up visits at 90±7 days (V2), 180±10 days (V3), 270±10 days (V4), 360±15 days (V5), 450±15 days (V6), and a final visit at 540±15 days (V7). The baseline visit and V3, V5, and V7 visits will be conducted face-to-face at the study center, whereas V2, V4, and V6 visits will be conducted by telephone.

At the screening visit (V0), which will take place within 60 days (8 weeks) prior to the baseline visit, the following activities will be performed: assessment of the inclusion criteria, obtaining informed consent after a full explanation of the objectives and characteristics of the study, randomization with assignment of the participant selection number, and scheduling an appointment for an ^18^F-flutemetamol positron emission tomography (^18^F-flutemetamol PET) scan.

The baseline visit (V1) involves assessing various study variables. The initial visit will take place on 3 days: on day 1, psychological tests will be administered to determine whether the individual meets the inclusion criteria. On day 2, the patients who pass the psychological tests will be analyzed (PET) for Aβ deposition in the brain. Those who are positive will pass the final psychological tests on day 3. The 3 days will not be more than 15 natural days apart. On the first day, the patient’s eligibility criteria and results of the ^18^F-flutemetamol PET scan will be checked. Data recorded will include demographics (age and sex), body weight, BMI, tobacco use, alcohol consumption, comorbidities, and concomitant medications. A first battery of neuropsychological tests will be completed. The hierarchical order of neuropsychological tests at the baseline visit on day 1 includes the following: (1) Mini-Mental State Examination (MMSE) scale; (2) Geriatric Depression Scale-15 (short version GDS-15); (3) Clinical Dementia Rating–Sum of Boxes (CDR-SB) scale; (4) Integrated Alzheimer’s Disease Rating Scale (iADRS); (5) Alzheimer’s Disease Cooperative Study–Activities of Daily Living for Mild Cognitive Impairment (ADCS-ADL-MCI) scale; and (6) Disability Assessment for Dementia (DAD-E) scale.

The hierarchical order of neuropsychological tests at the baseline visit on day 2 includes the following: (1) TAVEC; (2) Trail Making test (parts A and B); (3) clock drawing test (CDT); (4) Rey-Osterrieth Complex Figure (ROCF) test; (5) digit span test-forward and backward; (6) verbal fluency test; and (7) Frontal Assessment Battery (FAB).

Other procedures include fasting blood sampling for hematological and biochemical tests, including biomarkers of dementia, and provision of the study product for the first 6 months (180 days) of supplementation. Patients will be clearly instructed on how to take the study product, the importance of adhering to the treatment, and how to strictly maintain their dietary and lifestyle habits throughout the study. They will also be advised not to make any changes to their concurrent medications.

At visit 2 (via telephone), AEs will be recorded, concomitant medications with potential treatment changes will be checked, and visit 3 will be scheduled for 180±10 days. At visit 3, the same procedures as those performed at visit 1 (except for neuropsychological testing) and at visit 2 will be carried out. In addition, the study product for the following 6 months (180 days) of treatment will be dispensed; capsules returned from those given at visit 1 will be counted to assess compliance; and visit 4 at 270±10 days will be scheduled. At visit 4 (via telephone), the same procedures as at visit 2 will be followed, and visit 5 will be scheduled for 360±14 days. At visit 5, the same procedures as at the baseline visit will be performed. The battery of neuropsychological tests will be administered following the same hierarchy as at the baseline visit (V1). Assessment of AEs and concomitant medication, provision of the study product for the final 6 months (180 days) of treatment, and collection and counting of returned capsules will be performed. Also, visit 6 at 450±15 days will be scheduled. At visit 6 (via telephone), the same procedures as at visits 2 and 4 will be followed.

The final visit (V7) at the end of the study, at 540±15 days, will include a complete assessment of patients, including medical history; the battery of all neuropsychological tests was administered following the same hierarchy as in the baseline visit (V1) and visit 5 (V5), blood sampling for laboratory tests, evaluation of AEs and concomitant medication, collection and counting of the study product, and performance of an ^18^F-flutemetamol PET scan. A summary of the study design and procedures is shown in Figure 1. An overview of the study is shown in Table 1.

**Figure 1.**
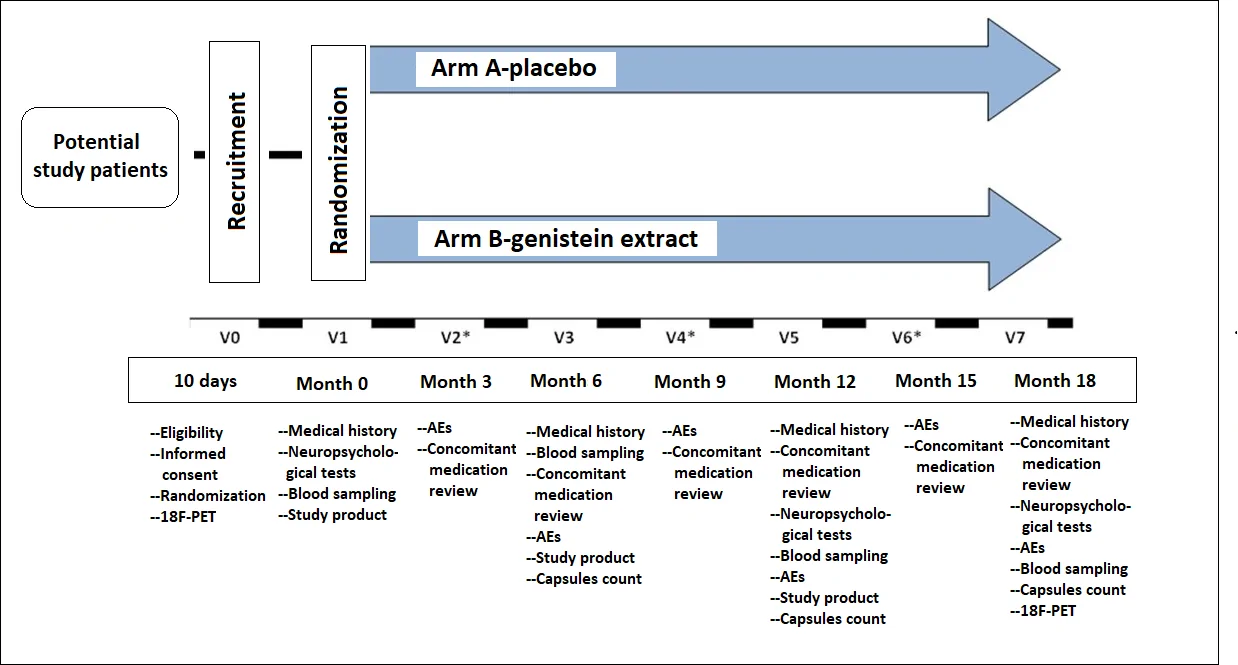
Study design and procedures. Asterisk denotes via telephone. ^18^F-PET: ^18^F-flutemetamol positron emission tomography; AEs: adverse events; V: visit.

**Table 1.** Overview of the study.

Procedures	V0^a^ (selection)	V1 (baseline)	V2 (telephone; (90 days)	V3 (180 days)	V4 (telephone; (270 days)	V5 (360 days)	V6 (telephone)	V7 (final visit; (540 days)
Eligibility, informed consent, and randomization	✓							
Neuropsychological tests		✓				✓		✓
PET^b^		✓						✓
Blood extraction		✓				✓		✓
Product delivery/return		Delivery		Both		Both		Return
Concomitant medication			✓	✓	✓	✓	✓	✓
Adverse events			✓	✓	✓	✓	✓	✓

a V: visit.

b PET: positron emission tomography.

### Variables and Data Collection

#### Neuropsychological Tests

##### Evaluation of Cognition Impairment, Activities of Daily Living, and Depression

The iADRS combines scores from 2 widely accepted measures, the Alzheimer’s Disease Assessment Scale–Cognitive Subscale (ADAS-Cog) [26] and the Alzheimer’s Disease Cooperative Study–Instrumental Activities of Daily Living (ADCS-iADL) [27]. The iADRS [27] is calculated as a linear combination of the total scores of the 2 components: the Alzheimer’s Disease Assessment Scale–Cognitive Subscale, 14-item version (ADAS-Cog_14_; score range 0-90) and the instrumental items of the ADCS-iADL (score range 0-56). Because higher scores on the ADAS-Cog_14_ reflect worse performance, whereas higher scores on the ADCS-iADL reflect better performance, the ADAS-Cog score is multiplied by (−1) in the calculation of the integrated scale. To anchor the ADAS-Cog at 0, a constant (90) is added. The iADRS score is then computed as the sum of the transformed ADAS-Cog_14_ and the ADCS-iADL, as shown in the formula below:


iADRS score=[−1(ADAS-Cog14)+90]+ADCS-iADL

The iADRS score ranges from 0 to 146, with lower scores indicating worse performance [28]. The iADRS takes 25 to 30 minutes to complete. The ADAS-Cog is validated in Spanish [29].

The CDR-SB [30] is a semistructured interview used to assess the global severity of cognitive impairment and functional decline in conditions such as Alzheimer disease. It involves interviewing both the patient and a caregiver to assess 6 domains: memory, orientation, judgment and problem-solving, community affairs, home and hobbies, and personal care. The CDR-SB total score ranges from 0 to 18, with the global severity of cognitive impairment categorized as 0: normal, 0.5: very mild (questionable cognitive impairment to very mild dementia), 1: mild (suggests mild dementia), 2: moderate (suggests moderate dementia), and 3: severe (suggests severe dementia) [31]. It takes 20 to 25 minutes to complete.

The ADCS-ADL-MCI [32] is a functional evaluation scale that assesses the ability of patients to perform activities of daily living (ADLs), with recall “in the past 4 weeks,” through a structured questionnaire administered to the informant/caregiver by a physician or qualified rater. The ADCS-ADL-MCI was derived from its parent measure, the ADCS-ADL, and adapted for patients with MCI. There are 2 forms of the ADCS-ADL-MCI, the 24-item and the 18-item versions. The 18-item ADCS-ADL-MCI [33], commonly used in clinical trials and clinical practice, is scored from 0 to 53 based on the patients’ degree of independence (from 0: fully dependent to 3: fully independent) in performing a wide range of activities, from basic personal care (eg, getting dressed, selecting one’s clothes, doing the laundry, making a meal, etc) to instrumental ADLs (eg, using a telephone, balancing the checkbox, writing notes, traveling out of home, etc). Higher ADCS-ADL-MCI scores indicate better functional abilities. It takes 15 to 25 minutes to complete.

The DAD-E [34] is a 40-item questionnaire designed to evaluate both basic and instrumental ADLs in patients with cognitive impairment. The scale is completed by a caregiver or proxy respondent, with recall “in the past 2 weeks.” Basic ADLs are dressing, hygiene, continence, eating, meal preparation, and telephoning, whereas instrumental activities include going out and outing, finance and correspondence, medications, and leisure and housework. The choice of answer is 1 point: yes (the person performs the activity without help or reminder); 0 points: no (the person performs the activity with some verbal or physical assistance from the caregiver); and N/A: not applicable (the person never did this activity or did not have the opportunity to do it in the past 2 weeks). The final score ranges from 0 to 80, with higher scores indicating greater functional independence and lower disability. A DAD score of 70 to 80 suggests no significant cognitive decline; a score of 60 to 70 indicates MCI; and a score <60 indicates mild to moderate dementia. The DAD instrument has been validated in Spanish [35] and takes 5 to 10 minutes to complete.

The GDS-15 is used to screen for depression in older adults and consists of 15 items that assess mental health based on feelings over the past week. Although it is not designed for the diagnosis of Alzheimer disease, it is useful to detect depressive symptoms that may coexist with dementia or affect quality of life. Examinees are asked to respond “yes” or “no” to each item in reference to the past week. One point is given for depressive responses. All responses are summed to calculate the final score, which ranges from 0 to 15. Scores ranging from 0 to 4 are classified as within normal limits, 5 to 9 as mild depressive symptoms, and 10 to 15 as moderate-to-severe [36]. A Spanish validated version of the GDS-15 scale is available [37]. It takes 5 to 10 minutes to complete.

The MMSE test is one of the most commonly used tools for the detection and evaluation of cognitive impairment, including Alzheimer disease. It is an 11-question measure that tests 5 areas of cognitive function: orientation (10 points), registration (3 points), attention and calculation (5 points), recall (3 points), and language and praxis (9 points). The maximum score is 30, with scores 27 to 30 indicating normal cognition, 24 to 26 indicating MCI, 20 to 23 indicating moderate impairment, and <20 indicating severe impairment. Since the original description in 1975 [38], the MMSE has been validated in many languages, including Spanish [39], and is extensively used in both clinical practice and research. Setting the upper limit at 30 addresses the MMSE’s ceiling effect, as individuals with high cognitive reserve or early-stage MCI can achieve normative scores (27/30) without ruling out actual cognitive impairment. Conversely, the lower limit of 22 allows for the detection of more advanced MCI or moderate dementia.

##### Evaluation of Episodic Memory and Delayed Recall

The TAVEC is the Spanish adaptation of the California Verbal Learning Test (CVLT) [40] and is widely used in Spanish-speaking populations [41]. It evaluates verbal episodic memory and learning capacity and comprises 5 phases: learning, interference, immediate recall, delayed recall, and recognition, using 2 different lists of 16 words (A and B). During the learning phase, a 16-word list (list A) is read aloud to the patients 5 times, and the number of words remembered is recorded. In healthy individuals, the expected total score is 40 to 50 words, whereas in Alzheimer disease, a score ≤30 may be expected. In the interference phase, another 16-word list (list B) is presented, and the number of words remembered and the scoring procedure are the same as in the learning phase. In the immediate recall phase, the participant is asked to recall the words from list A; normally, 8 to 10 words are remembered, and recalling ≤5 suggests difficulty in overcoming interference, as seen in Alzheimer disease. In the delayed recall phase, list B is used, and the same procedures as in the immediate recall phase are followed. In the final recognition phase, a list of 44 words is presented, including lists A and B and 12 new words (distractors), and the participant should identify words belonging to list A. The recognition phase is scored as the number of correct words minus the number of false positives (confusion with words that were not presented), with a score <0.7 suggesting Alzheimer disease. The TAVEC test takes 40 to 45 minutes to administer.

The ROCF test [42,43] involves the immediate copying of complex geometric shapes and a delayed recall (30 minutes). Scoring is based on a system of 18 elements that evaluates both the accuracy and organization of the drawing, with each element rated as 0 (absent or incorrect), 1 (partially correct), or 2 (correct), yielding a total score ranging from 0 to 36 points. Patients with Alzheimer disease may present difficulties in copying, reflecting impairments in visuospatial perception and visuo-constructive organization, as well as deficits in recalling, reflecting problems with visual memory and retention of information.

In the delayed recall phase of the ROFC test, 30 minutes after the initial copy, patients will be asked to reproduce the figure from memory, with performance scored using the same 18-element system as the immediate copy, evaluating both accuracy and organization. Moreover, the delayed recall and recognition phases of the TAVEC will also be repeated.

##### Evaluation of Executive/Attention Functions

The CDT will be used to quickly assess visuospatial and praxis abilities and may be useful for identifying both attentional and executive dysfunction [44]. Typically, patients are asked to draw a clock face, fill in all the numbers, and then set the hands to a specific time, most commonly “10 minutes after 11.” It takes 5 to 10 minutes to complete.

The Digit Span Test will be used in 2 formats: forward digit span and reverse digit span. Participants are presented with a random series of digits and are asked to repeat them in either the order presented (forward span) or in reverse order (backward span) [45]. Forward and backward formats rely on somewhat separable cognitive capacities: the simpler forward span task requires verbal working memory and attention, while the backward span task additionally tests cognitive control and executive function. Forward span scores typically range from 7 to 9 digits, whereas backward span scores range from 5 to 6 digits. Scores are usually lower in the context of Alzheimer disease. The test takes 5 to 10 minutes to complete.

The Trail Making test (parts A and B) involves visual scanning and working memory. Each part consists of 25 circles distributed over a sheet of paper [46]. In Part A, the circles are numbered 1 to 25, and the patient should draw lines to connect the numbers in ascending order, whereas in part B, the circles include both numbers (1-13) and letters (A-L) and the patient draws lines to connect the circles in an ascending pattern, but with the added task of alternating between the numbers and letters (ie, 1-A-2-B-3-C, etc). Results are reported as the time required to complete the task, with higher scores indicating greater impairment. Completion times for part A typically range from 40 to 60 seconds for cognitively healthy older adults, whereas part B averages between 60 and 90 seconds. Administration of the Trail Making test (parts A and B) takes 5 minutes.

The FAB is a brief tool that helps to assist in discriminating between dementias with a frontal dysexecutive phenotype and dementia of Alzheimer type [47]. It consists of 6 tests that correspond to each specific cognitive or behavioral domain related to the frontal lobes. The 6 domains are similarities (conceptualization), lexical fluency (mental flexibility), motor series (programming), contradictory instructions (sensitivity to interferences), Go/No-Go (inhibitory control), and prehension (grasp) behavior (environmental autonomy). Scores range from 0 to 18, with higher scores indicating better performance. Scores of 17 to 18 are considered normal, scores of 12 to 16 indicate mild impairment, scores of 9 to 11 indicate moderate impairment, and scores of 0 to 8 indicate severe impairment. A validated Spanish version of the FAB will be used [48]. The test takes 10 to 20 minutes to complete.

### ^18^F-Flutemetamol PET Imaging

Radioligands, such as ^18^F-flutemetamol, selectively bind to Aβ, allowing direct PET imaging of Aβ plaques in patients with Alzheimer disease [49]. Quantification of Aβ PET radioligand retention can detect changes in brain Aβ plaque burden over time. Amyloid-PET images will be acquired 90 minutes after the intravenous administration of the ^18^F-flutemetamol tracer (185 MBq). Standardized uptake values (SUVs) are commonly used as semiquantitative measures of Aβ burden, calculated by normalizing Aβ PET radiotracer retention in a target brain region of interest to a reference region not expected to accumulate amyloid. The SUVs from a set of selected brain regions are averaged to compute a composite SUV. Higher composite SUV values indicate a greater amyloid burden, and negative changes in the composite cortical SUV over time indicate decreases in cerebral amyloid burden.

### Serum Biomarkers

Evaluation of serum biomarkers can provide a more comprehensive perspective on underlying mechanisms and treatment response. Aβ peptides (Aβ1‐42 and Aβ1‐40), phosphorylated τ protein (p-tau181 and p-tau217), neurofilament light chain (NfL), and BDNF will be measured centrally at the laboratory facilities of “Pasqual Maragall Foundation” (Barcelona, Spain). Inflammatory markers, including C-reactive protein (CRP), IL-6, and TNFα, will be measured using analytical techniques available in the laboratories of the participating hospitals, following the manufacturers’ instructions when commercial kits are used.

### Safety Assessments

Safety outcomes will include spontaneously reported AEs, vital signs, physical examination, and results of laboratory tests, including blood cell count, liver function tests (aminotransferases [alanine aminotransferase (ALT) and aspartate aminotransferase (AST)], bilirubin, γ-glutamyl transpeptidase [GGT], lactate dehydrogenase [LDH]), renal function tests (blood urea nitrogen and creatinine clearance), and urinalysis.

### Evaluation Outcomes

The primary outcome of the study is the mean change in the iADRS score after 18 months of genistein consumption relative to baseline. The iADRS integrates global cognition results (ADAS-Cog 14) with results from activities of daily living (ADCS-ADL), thereby providing a comprehensive view of the patient’s cognitive and functional status.

Secondary outcomes include the mean changes in scores for the remaining neuropsychological tests at the end of the study relative to baseline, the reduction in amyloid plaque as assessed by ^18^F-flutemetamol PTC, and changes in biomarkers of Alzheimer disease. Neuropsychological tests prioritized as secondary outcomes were the MMSE, TAVEC, CDT, and ROCF, as these tests were evaluated in a previous study by our group [20].

### Statistical Analyses

#### Sample Size Estimation

The sample size was calculated based on the least-squares mean change in the iADRS score as the primary outcome of the study. Considering an SD of 1.56 for the least-squares mean change in the iADRS score at 76 weeks, obtained in a similar population of patients with Alzheimer disease [50], for a precision of 0.7 with an α level of 5% and statistical power of 80%, 61 patients per study group will be required, increasing to 82 patients per group assuming a 25% loss to follow-up.

#### Data Analysis

Analyzes will be performed across 2 datasets: the per-protocol population (ie, patients who completed the 18 month study period) and the intention-to-treat population (ie, all randomized patients who received at least one dose of the assigned treatment and attended at least one follow-up visit). Categorical variables are described as frequencies and percentages, and continuous variables as means, medians, SDs, 95% CIs, and ranges (minimum and maximum) for the overall study population and the experimental and placebo groups. To ensure comparability of baseline characteristics between the 2 study groups, categorical variables will be analyzed using the chi-square test or Fisher exact test, as appropriate, and continuous variables will be analyzed using the Student 2-tailed *t* test. Changes in study variables over the course of the study will then be evaluated using a repeated-measures ANOVA, with time as the within-participant factor, product (genistein or placebo) as the between-participant factor, and study site as a covariate. Tukey or Bonferroni correction procedures will be applied for post hoc analyses. The analysis of the intention-to-treat population will account for patients lost to follow-up. In the analysis of the per-protocol population, the last observation carried forward method will be used to handle missing data. Statistical significance is set at *P*<0.05. SPSS (version 21.0; IBM Corp) will be used to analyze the data. No interim analyzes are planned.

## Results

Funding for the study began in January 2024 and was provided by Trugon Investigaciones (AIE) through a grant to the Chair of Geroscience at Universidad Católica de Murcia. Data collection began in July 2026 and is projected to conclude in January 2029, consistent with the estimated 30-month study duration. Enrollment is expected to last approximately 9 months from the first study visit, which took place in July 2026. As of August 5, 2026, a total of 4 participants had been recruited. The follow-up period will extend for 18 months, with an estimated total study duration of 30 months. Data analysis has not begun and will start after the final participant completes follow-up, and the results of these analyses are expected to be published in late 2029.

## Discussion

### Principal Findings

MCI is a nosological entity proposed as an intermediate state between normal aging and dementia and frequently appears to represent an early stage of Alzheimer disease. However, the prognosis of MCI, the conversion risk to dementia, and the probability of stability or reversion are still under intense study. Data from a comprehensive systematic review and meta-analysis of 66 clinical-based studies, including a population of 21,822 participants with a diagnosis of MCI, reported a risk of conversion to dementia of 41.5% (95% CI 38.3%‐44.7%) [51]. Interestingly, the study highlights the need for specific MCI management, as follow-up length did not affect conversion risk, suggesting a slower or plateauing neurodegenerative process over time [50]. Thus, interventions that may improve cognition or delay the progression of cognitive decline represent a crucial step in the prognosis of mild neurocognitive disorders. Moreover, recent epidemiological data of Alzheimer disease alert the public health impact of the disease, with a number of 7.2 million Americans aged 65 years or older living with Alzheimer dementia today, and this number is expected to grow to 13.8 million by 2060 [52].

The present project aligns with the current research focus on Alzheimer disease: developing patient-centered, nonpharmacological interventions to slow its progression, which are crucial for managing symptoms, improving quality of life, and preserving independence. When developing the study, we paid particular attention to selecting the primary outcome. The iADRS is scientifically robust, has already been used in clinical trials of antiamyloid treatments for Alzheimer disease, and has been proven to be a sensitive and reliable tool capable of measuring the effectiveness of treatment on cognition and ADLs [50]. Moreover, the use of ^18^F-flutemetamol PET to monitor disease progression by demonstrating amyloid plaque load will help evaluate the effectiveness of genistein by tracking changes in amyloid deposition over time.

### Limitations

Although every effort will be made to administer the complete battery of psychoneurological tests at visits 5 and 7 (360 and 540 d after study initiation), the large number of assessments makes it difficult to ensure complete adherence to this procedure. To mitigate this potential limitation, the importance of completing the full battery of tests will be emphasized during the preceding telephone visits (visits 4 and 6).

### Conclusions

The proposed trial will be the first to evaluate the effects of oral supplementation with a natural extract of genistein on the progression of cognitive decline and activities of daily living in patients diagnosed with MCI due to Alzheimer disease, based on an extensive battery of neuropsychological tests, the assessment of amyloid plaques by neuroimaging, and measurements of serum levels of Alzheimer disease biomarkers. The observation of beneficial changes in the study variables after 18 months of genistein supplementation would be highly relevant from a clinical perspective, given the overwhelming health care and societal burden of dementia and the fact that genistein is an inexpensive and readily available natural compound. So far, however, the favorable effects of genistein in MCI remain preliminary, and the primary aim of this clinical trial is to determine whether genistein is capable of delaying the progression of cognitive decline in patients with MCI.

## Supplementary material

10.2196/98087 Checklist 1 SPIRIT checklist.

## Abbreviations

ADAS-Cog: Alzheimer’s Disease Assessment Scale–Cognitive Subscale
ADAS-Cog_14_: Alzheimer's Disease Assessment Scale–Cognitive Subscale, 14-item version
ADCS-ADL-MCI: Alzheimer’s Disease Cooperative Study–Activities of Daily Living for Mild Cognitive Impairment
ADCS-iADL: Alzheimer’s Disease Cooperative Study–Instrumental Activities of Daily Living
AE: adverse event
AIE: Trugon Investigaciones
ALT: alanine aminotransferase
ApoE: apolipoprotein E
AST: aspartate aminotransferase
Aβ: amyloid β
AβPP/PS1: Aβ precursor protein/presenilin 1 double transgenic
BACE1: β-secretase
BDNF: brain-derived neurotrophic factor
CDR-SB: Clinical Dementia Rating–Sum of Boxes
CDT: clock drawing test
CONSORT: Consolidated Standards of Reporting Trials
CRP: C-reactive protein
CVLT: California Verbal Learning Test
DAD-E: Disability Assessment for Dementia
FAB: Frontal Assessment Battery
GDS: Geriatric Depression Scale
GGT: γ-glutamyl transpeptidase
GMP: Good Manufacturing Practice
GSK-3β: glycogen synthase kinase-3β
iADRS: Integrated Alzheimer’s Disease Rating Scale
IL-6: interleukin-6
LDH: lactate dehydrogenase
MCI: mild cognitive impairment
MMSE: Mini-Mental State Examination
NF-kB: nuclear factor-kB
NfL: neurofilament light chain
NMDA: *N*-methyl-d-aspartate
p-tau: phosphorylated τ
PET: positron emission tomography
PI: principal investigator
PPARγ: peroxisome proliferator-activated receptor-γ
ROCF: Rey-Osterrieth Complex Figure
SOD2: superoxide dismutase 2
SPIRIT: Standard Protocol Items: Recommendations for Interventional Trials
STZ: streptozotocin
SUV: standardized uptake value
TAVEC: Aprendizaje Verbal España-Complutense Test
TNFα: tumor necrosis factor-α
UCAM: Catholic University of Murcia
